# Tumor growth accelerated by chemotherapy-induced senescent cells is suppressed by treatment with IL-12 producing cellular vaccines

**DOI:** 10.18632/oncotarget.10712

**Published:** 2016-07-19

**Authors:** Jana Simova, Olena Sapega, Terezie Imrichova, Ivan Stepanek, Lenka Kyjacova, Romana Mikyskova, Marie Indrova, Jana Bieblova, Jan Bubenik, Jiri Bartek, Zdenek Hodny, Milan Reinis

**Affiliations:** ^1^ Immunology Unit, Czech Centre for Phenogenomics, BIOCEV and Department of Transgenic Models of Diseases, Institute of Molecular Genetics of the ASCR, v.v.i., Prague 14220, Czech Republic; ^2^ Department of Genome Integrity, Institute of Molecular Genetics, v.v.i., Academy of Sciences of the Czech Republic, Prague 14220, Czech Republic; ^3^ First Faculty of Medicine, Charles University in Prague, Prague 12000, Czech Republic; ^4^ Danish Cancer Society Research Center, Copenhagen DK-2100, Denmark; ^5^ Department of Medical Biochemistry and Biophysics, Science For Life Laboratory, Division of Translational Medicine and Chemical Biology, Karolinska Institute, 17121 Solna, Sweden

**Keywords:** cellular senescence, cancer chemotherapy, docetaxel, IL-12, cell therapy

## Abstract

Standard-of-care chemo- or radio-therapy can induce, besides tumor cell death, also tumor cell senescence. While senescence is considered to be a principal barrier against tumorigenesis, senescent cells can survive in the organism for protracted periods of time and they can promote tumor development. Based on this emerging concept, we hypothesized that elimination of such potentially cancer-promoting senescent cells could offer a therapeutic benefit. To assess this possibility, here we first show that tumor growth of proliferating mouse TC-1 HPV-16-associated cancer cells in syngeneic mice becomes accelerated by co-administration of TC-1 or TRAMP-C2 prostate cancer cells made senescent by pre-treatment with the anti-cancer drug docetaxel, or lethally irradiated. Phenotypic analyses of tumor-explanted cells indicated that the observed acceleration of tumor growth was attributable to a protumorigenic environment created by the co-injected senescent and proliferating cancer cells rather than to escape of the docetaxel-treated cells from senescence. Notably, accelerated tumor growth was effectively inhibited by cell immunotherapy using irradiated TC-1 cells engineered to produce interleukin IL-12. Collectively, our data document that immunotherapy, such as the IL-12 treatment, can provide an effective strategy for elimination of the detrimental effects caused by bystander senescent tumor cells *in vivo*.

## INTRODUCTION

Cellular senescence, an irreversible cell-cycle arrest, represents a principal barrier against tumorigenesis [1–3]. Senescent cells do not proliferate, however, they remain metabolically active and are able to further influence biological processes within the body [4]. These ‘bystander’ effects are mediated by numerous molecules produced by the senescent cells, commonly termed senescence-associated secretory phenotype (SASP; [5, 6]). SASP comprises mainly growth factors, extracellular matrix components and remodeling factors, cytokines and chemokines including proinflammatory species as well as other factors mediating intercellular communications [7]. Besides cell death, senescence can be induced in tumor cells during chemotherapy or radiotherapy [8, 9]. In such case, the fate and the function of surviving senescent tumor cells, including the effects of SASP on tumor microenvironment, are considered to play an important role in tumor progression with influence on the effectiveness of antitumor therapy. There is accumulating evidence that the presence of senescent cells in the tumor and their SASP-related effects can be tumor growth-promoting [10]. It can be presumed therefore that not only the induction of cancer cell senescence itself, but also subsequent elimination of senescent cells will contribute, in a tumor-stage-dependent manner, to the overall anti-tumor barrier capacity. Indeed, it has been shown that oncogene-induced senescent murine hepatocytes were cleared by the immune system, through a process termed ‘senescence surveillance’ [11]. Importantly, impaired senescence surveillance in this model resulted in the induction of hepatocarcinoma. Furthermore, in MMTV-Wnt1-driven mammary tumors, persistence of senescent cells secreting senescence-associated cytokines displayed protumorigenic potential and contributed to tumor relapse [12]. The importance of senescence surveillance in aging has also been demonstrated in a study in which clearance of senescent cells (p16^Ink4a^) suppressed and/or delayed development of ageing-associated symptoms in a progeroid mouse model [13].

The first example documenting tumor-promoting effects of senescent cells co-administered with proliferating tumor cells was reported by Krtolica et al. [14] who showed that co-injection of senescent fibroblasts with human mammary tumor cells accelerated tumor growth in immunocompromised mice. Later on, however, it was shown that although senescent human prostate cancer cells increased proliferation of co-cultured tumor cells *in vitro,* such accelerating impact on xenograft tumor cell growth in nude mice was not observed [8] indicating impact of some still unidentified factors on this phenomenon.

Interleukin 12 (IL-12), a cytokine connecting innate and adaptive immunity, represents one of the important players in induction of anti-tumor immune response [15]. Produced mainly by antigen presenting cells, such as dendritic cells, macrophages, monocytes or B cells upon their activation, IL-12 exerts its effects mainly through induction of IFNγ, as well as NK and T cell activation [16, 17]. Antitumor immunotherapy with IL-12 administered in different forms, including the usage of irradiated tumor cells producing IL-12, has been studied [15, 18, 19]. In several experimental tumor models, including those used in our laboratory, anti-tumor immunogenicity could be enhanced by administration of IL-12 or by gene therapy with tumor cells engineered to produce IL-12 (for reviews, see [20–22]).

This intriguing accumulating data inspired our present working hypothesis, namely that IL-12-based immunotherapy might be able to mitigate or entirely eliminate the pro-tumorigenic effects of bystander senescent cells. Indeed, here we document an acceleration of tumor growth, when proliferating TC-1 tumor cells were co-administered into syngeneic mice together with syngeneic tumor cells that had been subjected to senescence-inducing treatment with docetaxel (DTX). Furthermore, we also document effective treatment of such tumors by cell therapy using irradiated IL-12-producing tumor cells.

## RESULTS

### DTX induces senescence in mouse tumor cells TC-1 and TRAMP-C2

**Figure 1:**
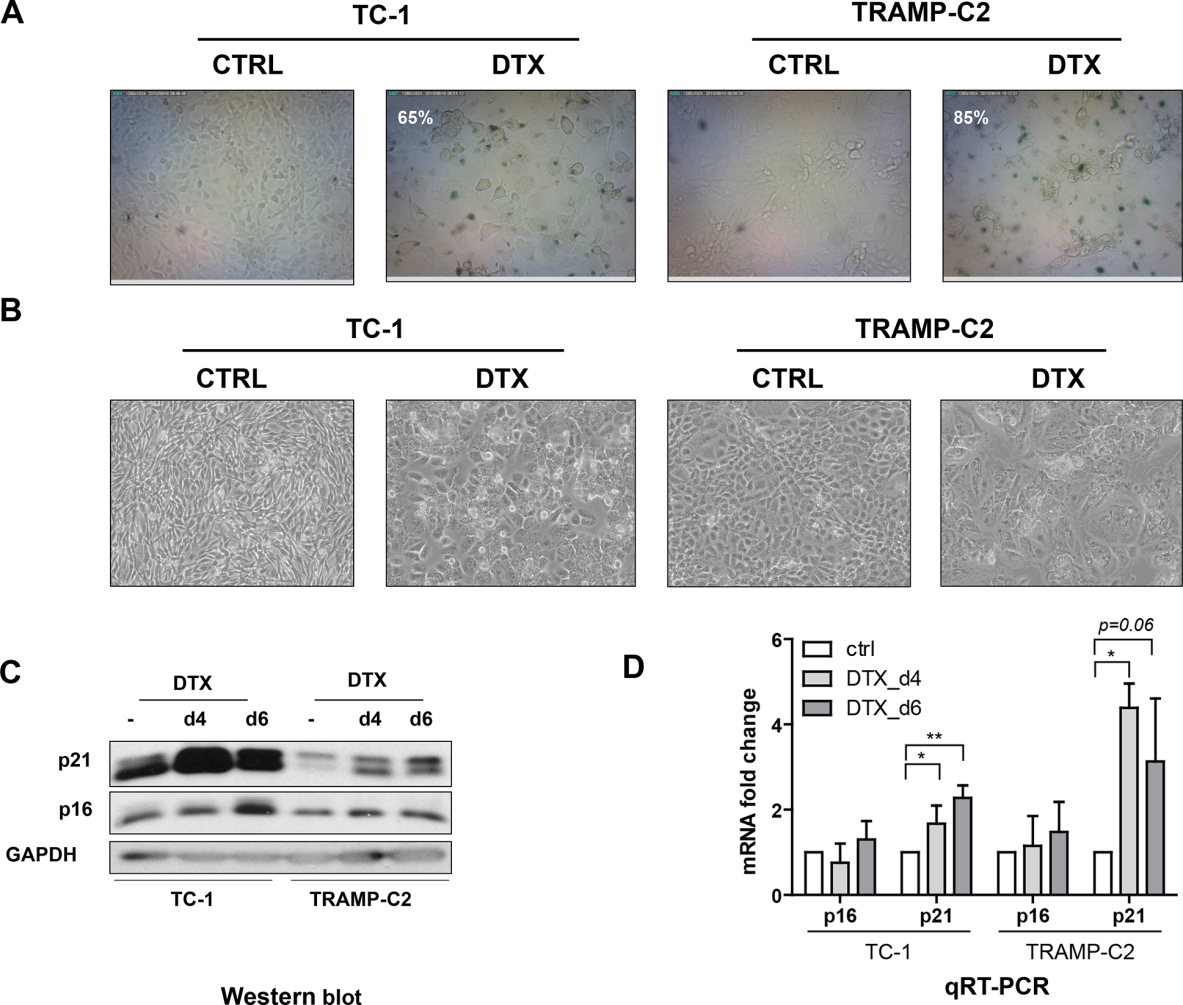
Docetaxel induces senescence in TC-1 and TRAMP-C2 cells Senescence-associated β-galactosidase activity in TC-1 and TRAMP-C2 cells treated with DTX (7.5 μM) for 4 days (**A**). Phase contrast microscopic images of control and DTX-treated (7.5 μM) TC-1 and TRAMP-C2 cells at day 4 after the treatment (**B**). Immunoblotting detection of mouse p16INK4A (p16) and p21waf1/cip1 (p21) in control and DTX-treated (7.5 μM) TC-1 and TRAMP-C2 cells harvested at day 4 and 6 after the treatment. GAPDH was used as a loading control (**C**). qRT-PCR quantification of p16 and p21 in control and DTX-treated (7.5 μM) TC-1 and TRAMP-C2 cells harvested at day 4 and 6 after the treatment. Data represent means ± S.D. *p < 0.05, **p < 0.005 (**D**).

**Figure 2:**
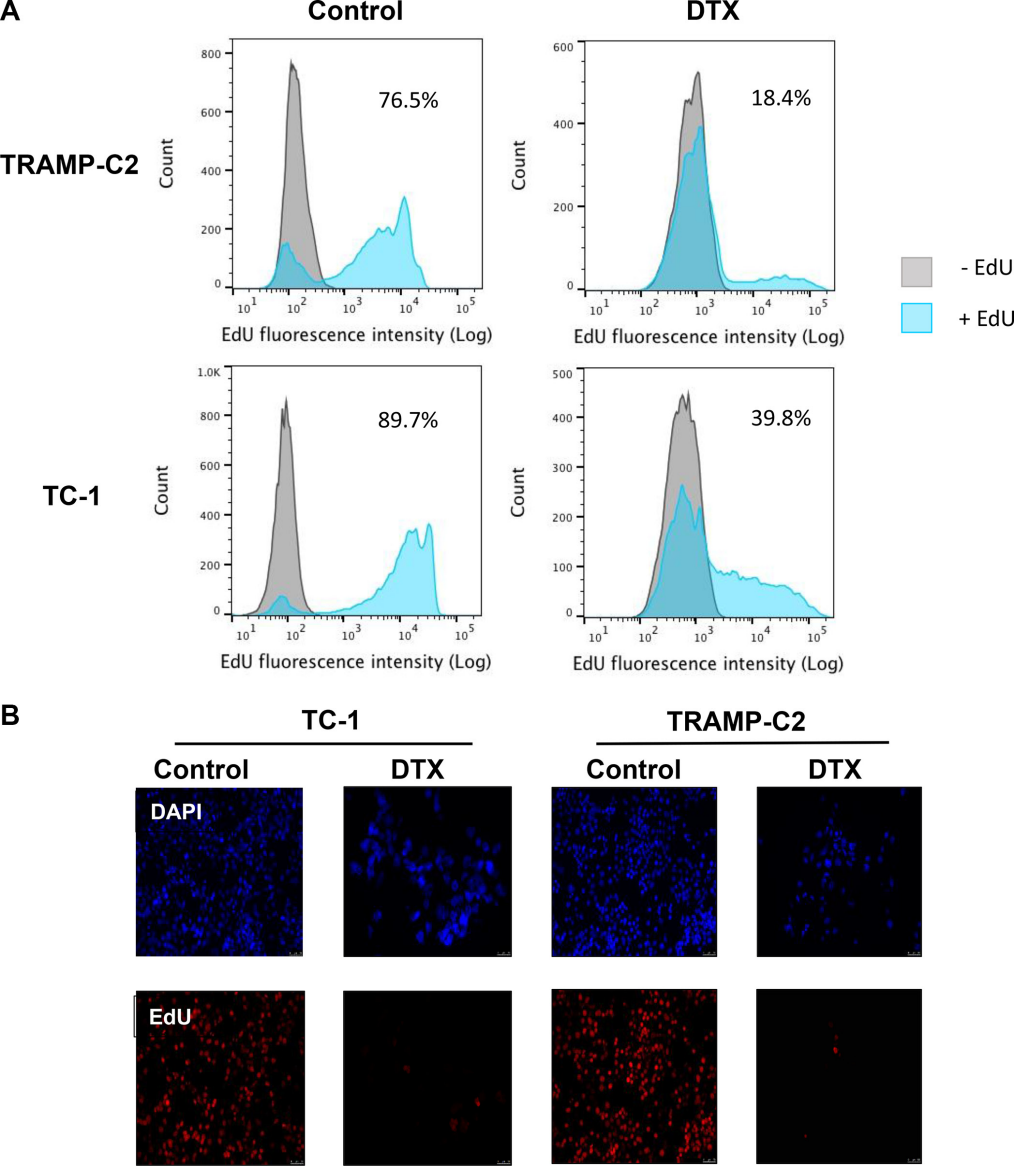
Analysis of TC-1 and TRAMP-C2 cell proliferation by EdU incorporation The cells were driven to senescence by 4-day treatment with 7.5 μM docetaxel (DTX) and then incubated with 10 μM EdU for 6 h. Click-iT reaction was performed on fixed cells and FACS analysis was carried out to determine the fraction of proliferating cells in DTX-treated and control samples (**A**). Control and DTX-treated TC-1 and TRAMP-C2 cells were incubated with 10 μM EdU for 6 h. Following fixation, Click-iT reaction was performed and the coverslips were mounted with Mowiol containing DAPI (**B**).

**Figure 3:**
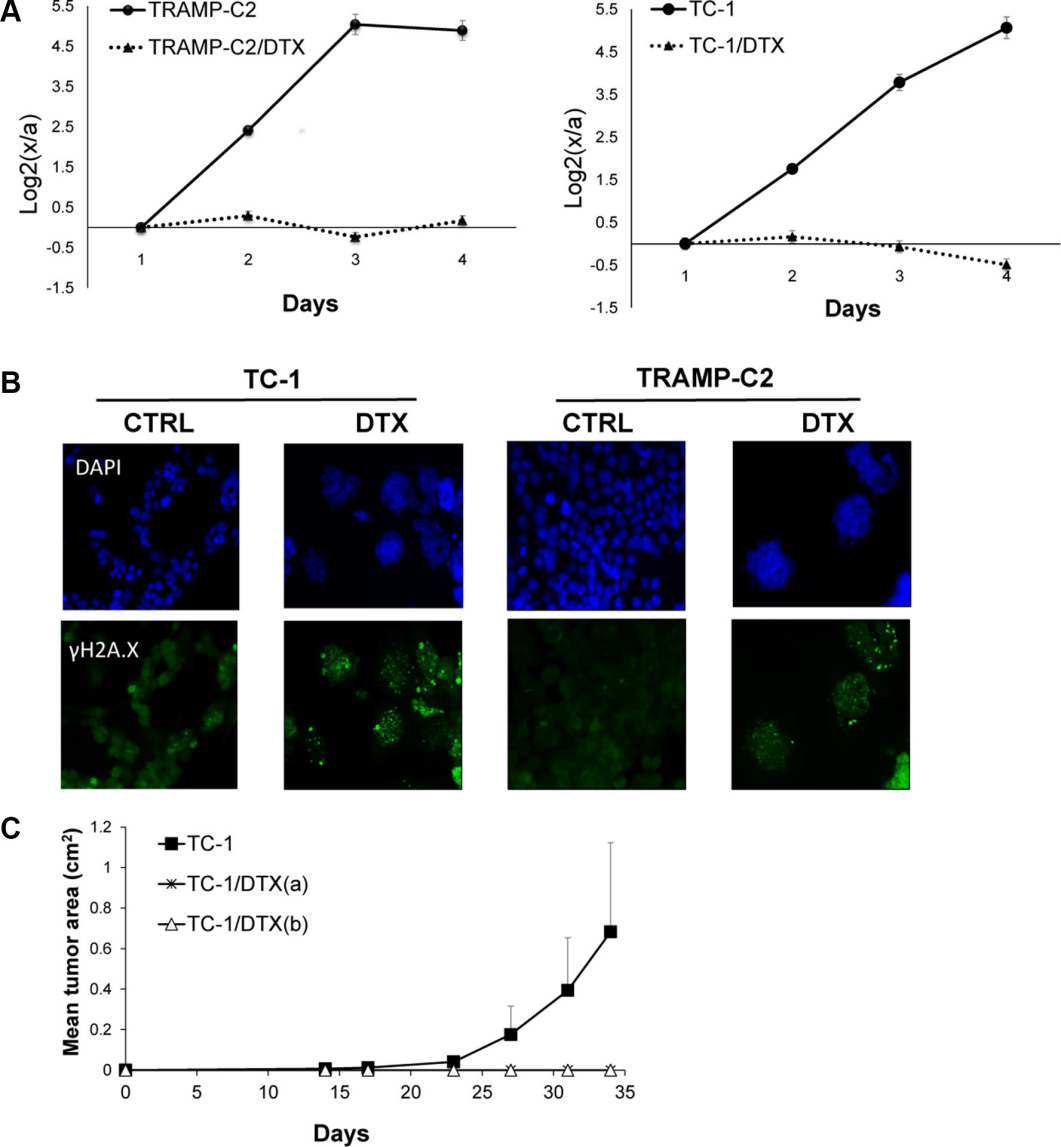
Docetaxel-induced DNA damage and senescence in TC-1 and TRAMP-C2 cell lines *in vitro* and *in vivo* Cells were seeded onto 6-well plates in triplicates and treated with 7.5 μM docetaxel (DTX) or left untreated (control). Cell proliferation was determined by counting cell number every 24 h and plotted as log_2_ ratio of final cell number to number of seeded cells, to compensate for differential seeding capacity of control versus the DTX-treated cells (**A**). To detect DNA damage, control and DTX-treated cells were stained with phosphoSer139 H2AX (γH2AX) antibody and mounted with Mowiol containing DAPI (**B**). Mice were transplanted s.c. on day 0 with TC-1 cells (3 × 10^4^) or with senescent DTX-treated cells at the doses 3 × 10^4^ [TC-1/DTX(a)] or 5 × 10^5^ [TC-1/DTX(b)] tumor cells, respectively, and the tumor growth was monitored. The experiment was repeated three times with similar results (**C**).

First, we evaluated the impact of DTX in terms of senescence induction, using two C57Bl/6 mice-derived tumor cell lines TC-1 and TRAMP-C2 of lung and prostate epithelial origin, respectively. Both TC-1 and TRAMP-C2 cells were susceptible to DTX and underwent senescence after a four-day incubation with 7.5 μM DTX [23]. After this treatment, the vast majority of TC-1 and TRAMP-C2 cells were alive but senescent, as characterized by the lack of cell proliferation, increased senescence-associated-β-galactosidase activity, characteristic cell morphology and increased expression of p16INK4a and p21waf1 inhibitors of cyclin-dependent kinases. Most of the senescent cells showed persistent DNA damage response, as judged from the presence of DNA damage foci positive for serine 139-phosphorylated histone H2AX (γH2AX; Figure 1, Figure 3B). Cessation of DNA replication was verified by incorporation of EdU. Only limited subsets of EdU-positive cells were observed in both TC-1 and TRAMP-C2 cell populations by FACS analysis (Figure 2A). Such residual EdU positivity can most likely be accounted for by ongoing DNA repair of the observed DNA damage (γH2AX) and/or aberrant endoreduplication uncoupled from cell division (Figure 2B) as we did not observe any proliferation of cells upon the DTX-treatment (Figure 3A). Most importantly for our subsequent experiments, subcutaneous administration of such senescent cells into animals did not lead to development of tumors (Figure 3C).

Pro-tumorigenic effects of senescent cells are believed to be mediated through their secretome [5]. Therefore, we analyzed any potential expression changes among 84 cytokines/chemokines evaluated during the course of the DTX-induced senescence in TC-1 and TRAMP-C2 cell lines. Indeed, expression of more than two dozens of cytokines was altered (either upregulated or downregulated; fold change > 2) in both TC-1 and TRAMP-C2 tumor cell lines. Elevated mRNA levels of a number of inflammatory and immunoregulatory factors, such as CSF2, CXCL1, CXCL16, TNFSF13b, adiponectin, CCL19, CCLl20, CCL4, CCL5, CD70, CNTF, CSF3, CXCL10, HC, IL-10, IL-13, IL-17f, IL-27, IL-6, LIF, TNF were observed (Supplementary Table 1). Notably, some of these factors are endowed with documented pro-tumorigenic properties, such as CXCL1 or IL-6 [7]. Elevated mRNA levels of the TNF family (FasL, TNSFS13b, TNF-α) were observed as well.

### Admixture of senescent cells accelerates tumorigenic potential of proliferating TC-1 cells

**Figure 4:**
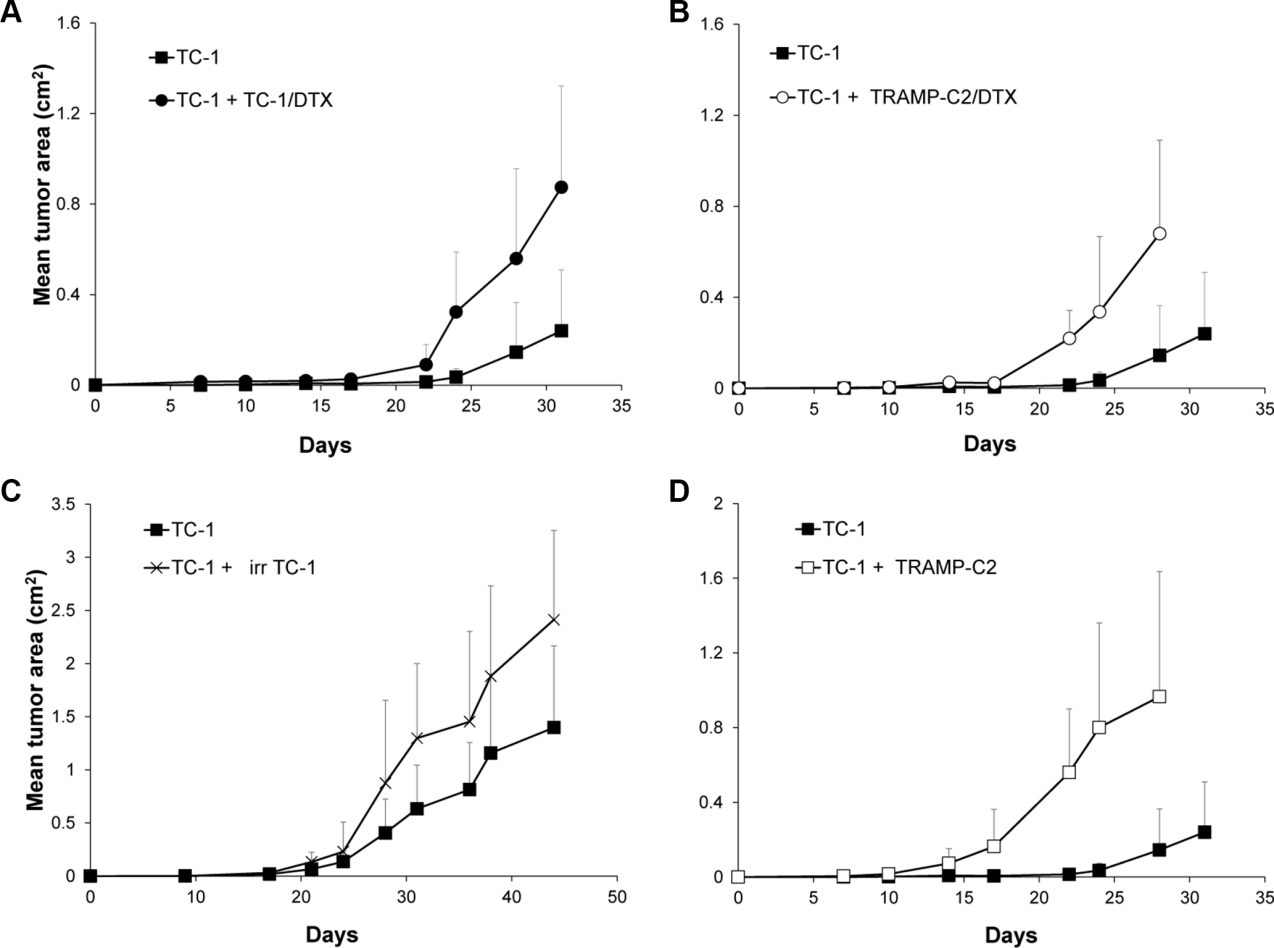
Co-administration of DTX-treated senescent cells accelerates tumor growth Mice were transplanted s.c. on day 0 with TC-1 cells (3 × 10^4^ cells per mouse) admixed with senescent, DTX-treated TC-1 cells (TC-1/DTX; 3 × 10^5^) (**A**); with senescent, DTX-treated TRAMP-C2 cells (TRAMP-C2/DTX; 3 × 10^5^ cells per mouse) (**B**); with irradiated TC-1 cells (150 Gy; 3 × 10^5^ cells per mouse) (**C**); or proliferating TRAMP-C2 cells (3 × 10^5^) (**D**). Controls were mice transplanted s.c. on day 0 with proliferating TC-1 cells (3 × 10^4^ cells per mouse) only. In all experiments, significant acceleration of the tumor growth was observed (*p* < 0.05). The experiments were repeated three times with similar results.

**Figure 5:**
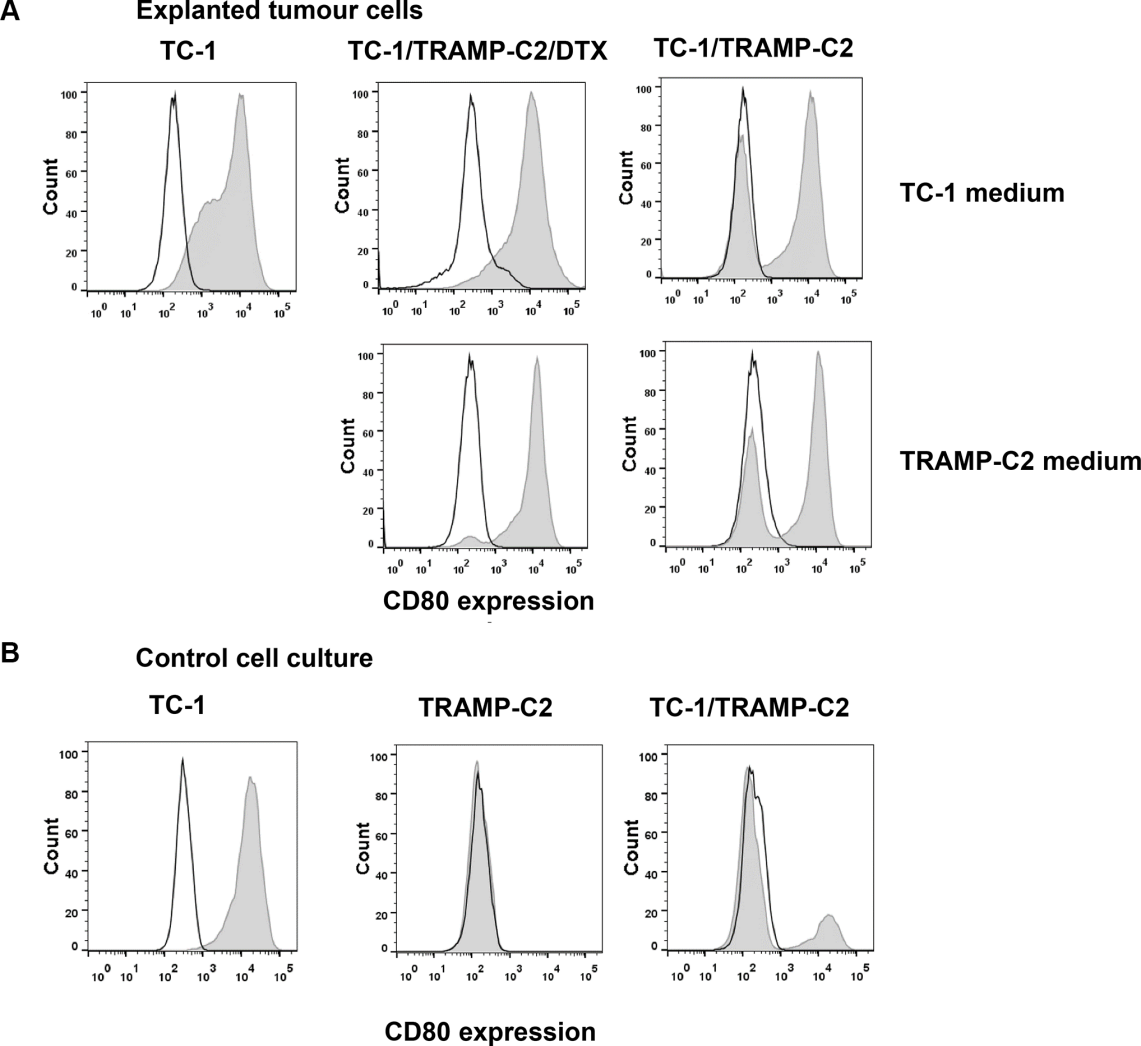
Cell surface marker analysis of explanted tumor cells Explanted tumor cells were cultured for seven days and analyzed for the proportion of TC-1 (CD80^+^) and TRAMP-C2 (CD80^−^) cells by flow cytometry using CD80 as a marker (filled histograms; empty histograms represent isotype controls). To exclude leukocytes from the analysis, CD45^+^ cells were gated out. For comparison, explanted cells were cultured in the culture medium optimized either for TC-1 cells (upper row) or for TRAMP-C2 cells (lower row) (**A**). Flow cytometry analysis of the CD80 cell surface expression on cultured TC-1 and TRAMP-C2 cells, as well as their mixed culture (**B**).

In order to test whether ‘bystander’ senescent cells can influence tumor growth, proliferating TC-1 tumor cells were injected into mice either alone (3 × 10^4^ cells/mouse) or admixed with docetaxel-induced senescent TC-1 cells (3 × 10^5^ cells/mouse). As shown in Figure 4A, co-administration of the bystander DTX-treated (senescent) cells and proliferating TC-1 cells into syngeneic animals resulted in significantly accelerated tumor growth, as compared to the tumors when the proliferating TC-1 cells were injected alone. Next, we performed an analogous experiment in which TC-1 proliferating cells were co-administered with senescent TRAMP-C2 cells (Figure 4B). Again, the presence of syngeneic bystander senescent cells accelerated the tumor growth. Significantly accelerated tumorigenicity was achieved also with admixture of proliferating TRAMP-C2 cancer cells (Figure 4C), as expected, and, interestingly, by adding lethally irradiated tumor cells (Figure 4D). Flow cytometry phenotypic analysis of cells explanted from the tumors and cultured for seven days *ex vivo*, taking advantage of the TC-1 and TRAMP-C2 mutual discrimination using the CD80 cell surface marker (TC-1 cells are CD80-positive while TRAMP-C2 are CD80-negative), revealed that the vast majority of tumor cells present in the tumors that had arisen from co-injection of TC-1 proliferating cells with senescent TRAMP-C2 cells were TC-1 tumor cells (Figure 5). This indicates that the senescent cells did not bypass senescence *in vivo* in our settings and thus they did not contribute to the growing cell mass of the tumor. On the other hand, admixture of proliferating instead of senescent TRAMP-C2 cells resulted in tumors consisting of both TC-1 and TRAMP-C2 cells, in spite of the fact that the TRAMP-C2 cells were injected in a number lower than the number of cells sufficient to ensure tumor growth in all injected animals. Collectively, these data demonstrate that bystander senescent tumor cells can evoke a tumor growth-promoting microenvironment, a property shared by bystander lethally irradiated tumor cells.

### IL-12-based immunotherapy suppresses senescence-accelerated tumor growth

**Figure 6:**
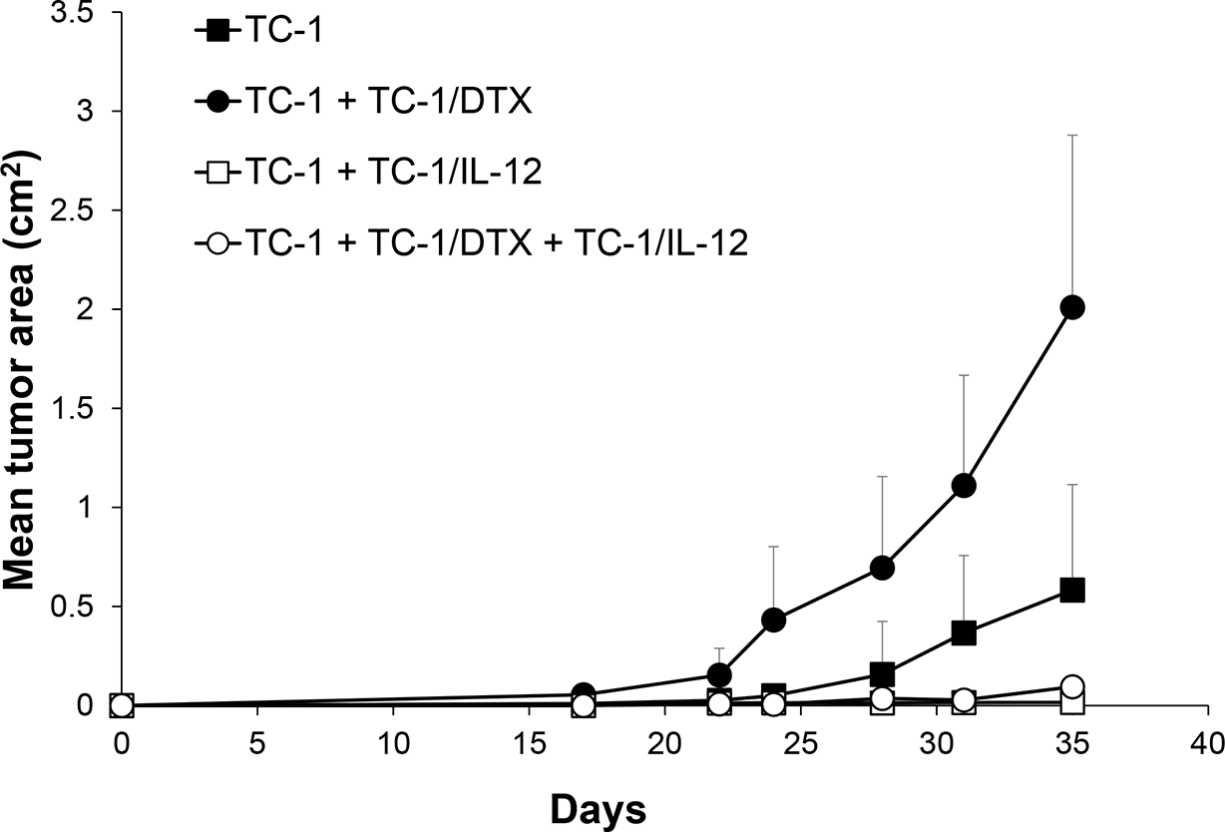
IL-12-based immunotherapy suppresses senescence-accelerated tumor growth Mice were transplanted on day 0 s.c. with TC-1 cells (3 × 10^4^), docetaxel-induced senescent TC-1/DTX (3 × 10^5^) or TC-1 cells (3 × 10^4^) admixed with TC-1/DTX. IL-12 producing TC-1/IL-12 cells were administered on day 3 in the vicinity of transplanted control TC-1 cells and TC-1 cells admixture with TC-1/DTX senescent cells. The experiment was repeated twice with similar results. *p* < 0.05 TC-1 as compared to TC-1/DTX; *p* < 0.05 TC-1 as compared to TC-1 + TC-1/IL-12; *p* < 0.05 TC-1 + TC-1/DTX vs. TC-1 + TC-1/DTX + TC-1/IL-12.

We have previously reported induction of IFNγ upon *in vivo* administration of an IL-12-producing cellular vaccine by ELISPOT analysis of spleen cells, as well as determination of the IFNγ levels in plasma by ELISA [24]. Given that IL-12-mediated antitumor effects are associated with induction of IFNγ, we have hypothesized that immunotherapy with IL-12 could be effective against both the proliferating and senescent tumor cells by concomitantly inducing non-specific as well as specific immune responses and thereby inhibiting growth of the senescent cell-accelerated tumors. To test this working hypothesis, here we employed irradiated IL-12-producing TC-1-derived cells, injected into the vicinity of *in vivo* growing tumors in two scenarios: i) either tumors growing from proliferating TC-1 cells alone, or ii) tumors growing in an accelerated fashion upon co-injection of the proliferating TC-1 cells with TC-1/DTX senescent cells. Consistent with our hypothesis, compared to rapid growth of mock-treated tumors, treatment with the IL-12-producing TC-1-based cellular vaccine greatly inhibited tumor growth under both tumorigenic scenarios (Figure 6). We conclude from these *in vivo* experiments that IL-12-based immunotherapy strategies analogous to the one we evaluated in this pre-clinical study may be effective also against the accelerated tumor growth fueled by the local microenvironment modified by the presence of bystander tumor senescent cells.

## DISCUSSION

From a broader perspective, our present results contribute to the field of tumor cell senescence and immunotherapy in several ways.

First, we show that chemotherapy-induced (here by DTX treatments) tumor cell senescence can indeed exert a tumor-promoting effect under syngeneic *in vivo* conditions. DTX, a chemotherapeutic agent used either alone or in standard-of-care combination treatments for various types of human malignancies, has been previously shown to induce massive cellular senescence in several tumor cell lines [23, 25]. In this study, we have demonstrated that DTX, when administered to two tumorigenic cell lines TC-1 and TRAMP-C2 *in vitro* in subapoptotic doses, induced persistent DNA damage response, cell cycle arrest and development of senescence-like phenotype accompanied by complex alteration of cytokine expression/secretion. This is in agreement with the current views that DNA damage signaling and repair networks are closely tied with the immune response signaling pathways [26]. Dependent on (patho)physiological context, such links can have either positive or detrimental impact on organismal health. Although pro-tumorigenic effects of senescent cells, namely fibroblasts, mediated by their SASP have been studied in the past [6], there has been paucity of studies documenting pro-tumorigenic effects of tumor cells brought into senescence by genotoxic chemotherapeutic agents, a gap in our knowledge partly filled by our present results.

Second, our major objective was to set up an *in vivo* model in which the impact of senescent cells on tumor growth could be investigated in the context of non-immunocompromised syngeneic animals, to better mimic the clinical human scenario. We argued that such model would help elucidate the biological impact of bystander senescent tumor cells under conditions permissive for interactions of the growing tumor with the host immune system, a complex interplay that can dramatically influence tumor microenvironment and cancer development in general. This fundamental biological aspect of tumorigenesis is unfortunately missing in the widely used xenograft tumor models based on immunodeficient animals, and it is a major advantage of the dataset presented here.

Another major advantage of our present study is the nature of the senescent cells used in our *in vivo* experiments. Thus, most previous studies dealt with the effects of senescent fibroblasts present in the tumor stroma on the proliferation of human tumor cells. Bavik et al. have demonstrated that both direct co-culture and conditioned medium from senescent fibroblasts stimulated neoplastic epithelial cells proliferation *in vitro* [27]. Further, senescent fibroblasts have been shown to promote neoplastic transformation of partially transformed ovarian epithelial cells in a three dimensional spheroid cell culture model [28]. Moreover, *in vivo* experiments were performed using nude mice in which xenograft tumor growth was monitored. Accelerated tumor growth was observed when senescent fibroblasts (1.5 × 10^6^ cells) were co-injected with the same number of proliferating MDA-231 human breast cancer cells into (*nu/nu*) mice [14]. However, in the study performed by Ewald et al., the admixture of 0.5 × 10^6^ senescent doxorubicin-treated DU-145 human prostate cancer cells with the equal number of proliferating cells did not result in any growth acceleration of the xenografted tumor [8]. In contrast, in our present study using syngeneic immunocompetent C57BL/6 model, we observed that the presence of senescent syngeneic tumor cells did augment the growth of tumors *in vivo*. We propose that the apparent discrepancy between the negative data of Ewald et al. [14] on the one hand, and our positive results documenting tumor growth acceleration on the other, might be attributable to better fitness and proliferation potential of tumor cells in the syngeneic system, cell type specific sensitivity or differences in the SASP of senescent cells or, importantly, the requirement of interactions between the senescent cells and the immune system or developing tumor stroma. Indeed, these important factors that are present in our syngeneic, immunocompetent model system, cannot be established in any heterologous system due to an uneven compatibility of mouse and human signaling components and compromised immunity. In addition, we believe that using much fewer senescent cells in our present experiments (we used the ratio between the proliferating tumor cells: 3 × 10^4^ cells/mouse, and the admixed docetaxel-treated senescent TC-1 or TRAMP-C2 cells at 3 × 10^5^ cells/mouse), compared to higher amounts of senescent cells in the previous studies [14], is a more realistic scenario that further supports the value of our model system.

Notably, our analysis of cytokine and chemokine transcript levels in the DTX-treated senescent tumor cells demonstrated upregulation of numerous cytokine species including those implicated in supporting tumor growth (such as CXCL1, IL-1 or IL-6). This effect of docetaxel is in accord with previous studies showing that persistent DNA damage response induced by various chemotherapeutics results in complex changes in cytokine expression and secretion (reviewed in [6]). As expected, when comparing cytokine profiles among different cell lines, there are some variations in the levels of specific cytokines. However, some shared, consistently altered species found in human senescent cells such as IL6 and CSF2 are also present in the secretome of the DXT-induced mouse senescent cells used here. For given tumor cells, these variations in secreted signaling ligands can have specific contributions to their respective tumor microenvironment and can possibly result in distinct responses to the same therapeutics.

In control experiments, we have proved that the growth acceleration could be achieved also by a senescent tumor cell type distinct from the proliferating one. This suggests that senescent cells can promote growth of tumor cells of different tissue origin, thus potentially fueling secondary malignancies of either the same or different tissues exposed to chemotherapy. Furthermore, relevant to radiotherapy and its potential late adverse effects, we show here in yet another set of experiments that also cells in which genotoxic stress was induced by irradiation, rather than chemotherapy, share the capacity to promote accelerated tumor growth.

There is still debate on ‘stability’ versus reversibility of the senescence phenotype, and there is mounting evidence that senescent cells can sometimes bypass their growth arrest and re-enter the cell cycle [29]. Interestingly, docetaxel-treated tumor cells can form polyploid senescent cells and subsequently re-enter the cell cycle and acquire a chemoresistant phenotype [30]. However, our analyses of the cell surface markers of the explanted tumor cells that formed the established tumors after TC-1 inoculation, the growth of which had been accelerated by the presence of senescent TRAMP-C2 cells, did not suggest any significant degree of senescence bypass among the TRAMP-C2 cells, since the vast majority of the explanted tumor cells were of TC-1 origin. These data suggest that the tumor growth was accelerated by paracrine pro-tumorigenic effects of the bystander senescent tumor cells. Unfortunately, in the scenario we have used, it is impossible to distinguish between direct effects on tumor cell proliferation versus more indirect impact through the tumor microenvironment or antitumor immunity, as the critical processes probably occurred shortly upon tumor cell administration when they are very difficult to follow. In a control experiment, in which TC-1 tumor cells were co-administered with proliferating TRAMP-C2 cells (although in suboptimal dose), analysis of cells from explanted tumors revealed that both TC-1 and TRAMP-C2 cells were present, as expected.

The major objective of our study was to develop a murine model that would enable us to test the immunotherapy efficacy in elimination of senescent cells and/or their detrimental tumor promoting effects and to demonstrate the effectiveness of the immunotherapy approach. Although senescent cells do not proliferate, they remain alive *in vitro* for long periods of time. Using a mouse liver carcinoma model, it has been demonstrated, that senescent tumor cells induced innate immune response due to their inflammatory cytokine secretion, accompanied by increase of leukocyte attracting molecules like ICAM1 or VCAM1. Consequently, senescent tumor cells were cleared from the organism [31]. Senescent cells can also induce specific immunity, as has been illustrated through their use as experimental cellular vaccines [32]. These facts suggest that detrimental effects of senescent cells can be eliminated using effective immunotherapy. IL-12, as a cytokine bridging innate and adaptive immunity, capable of activating NK cells, as well as inducing Th1 immune responses, appears to be a suitable candidate for such treatment. We have previously demonstrated the potential of IL-12-producing cellular vaccines that can be conveniently administered in the tumor vicinity, in several therapeutic chemo-immunotherapeutic settings. Here, we advance such studies and clearly document that the IL-12-based therapy can effectively inhibit development of tumors, including those whose growth was accelerated by the presence of bystander senescent cells.

Collectively, our work presented here has established a murine model that is beneficial for research into pro-tumorigenic effects of senescent cells, as well as innovative therapeutic strategies. Last but not least, our present dataset has documented that IL-12-mediated therapy may represent a feasible way to minimize or eliminate the adverse tumor-promoting effects of bystander senescent cells that may accumulate *in vivo* either due to endogenous oncogene-induced stimuli or insults caused by genotoxic chemotherapy and radiotherapy.

## MATERIALS AND METHODS

### Cell culture

TC-1 cell line was obtained by *in vitro* co-transfection of murine lung C57BL/6 cells with HPV16 *E6/E7* and activated human *H*-*Ras* (G12V) oncogenes [33]. IL-12-gene modified TC-1/IL-12 (231/clone 15) cells used for immunotherapy produced *in vitro* 40 ng IL-12/1 × 10^5^ cells/ml medium/48 h and were irradiated (150 Gy) before use [34]. TC-1 cells were cultured in RPMI 1640 medium supplemented with 10% fetal calf serum, L-glutamine and antibiotics. TRAMP-C2 prostate carcinoma cells [35] were obtained from ATCC collection. TRAMP-C2 cells were maintained in D-MEM medium (Sigma-Aldrich, Saint Louis, MO, USA) supplemented with 5% FCS, Nu-Serum IV (5%; BD Biosciences, Bedford, MA, USA), 5 mg/ml human insulin (Sigma-Aldrich), dehydroisoandrosterone (DHEA, 10 nM; Sigma-Aldrich) and antibiotics [36].

### Induction of senescence and characterization of senescent cells

To induce senescence in tumor cells, cells were cultured for 4 days in medium containing 7.5 μM DTX (Actavis, North Brunswick, NJ). Senescence was evaluated by using Senescence β-galactosidase Staining Kit (Cell Signaling Technology, Danvers, MA, USA) according to manufacturer's instructions. Images were captured by Dino-Lite (Dino-Lite Europe (Naarden, Netherlands) by using inverted tissue culture microscope Nicon TMS (Nicon, Tokyo, Japan) and by inverted tissue culture microscope Nicon Eclipse TE300 (Nicon, Tokyo, Japan) equipped with Leica DFC490 camera and LAS AF software (Leica Microsystems, Wetzlar, Germany).

### Antibodies

The following antibodies were used for immunoblotting: anti-mouse p21^waf1/cip1^ (p21) rabbit monoclonal antibody (ab109199) and anti-mouse p16INK4A (p16) rabbit polyclonal antibody (ab189034) were purchased from Abcam (Cambridge, UK), anti-mouse GAPDH rabbit monoclonal antibody (14C10) was purchased from Cell Signaling Technology (Danvers, MA, USA). IgG-HRP anti-rabbit secondary antibody produced in goat (170–6515) was purchased from Bio-Rad Laboratories (Hercules, CA, USA).

### SDS-PAGE and immunoblotting

Cells were washed with PBS, harvested into Laemmli SDS sample lysis buffer (2% SDS, 50 mM Tris-Cl, 10% glycerol in double distilled water) and sufficiently sonicated (3 × 15 seconds at 4 micron amplitude with 15 seconds cooling intervals) on Soniprep 150 (MSE, London, UK). Concentration of proteins was estimated by the BCA method (Pierce Biotechnology, IL, Rockford, USA). 100 mM DTT and 0.01% bromphenol blue was added to lysates before separation by SDS-PAGE (14% gel was used). The same protein amount (35 μg) was loaded into each well. Proteins were electrotransferred onto a nitrocellulose membrane using wet transfer and detected by specific antibodies combined with horseradish peroxidase-conjugated secondary antibody. Peroxidase activity was detected by ECL (Pierce Biotechnology). GAPDH was used as a marker of equal loading.

### Quantitative real time PCR (qRT-PCR)

Total RNA samples were isolated using RNeasy Mini Kit (Qiagen Sciences, Germantown, MD, USA). First strand cDNA was synthesized from 500 ng of total RNA with random hexamer primers using High-Capacity cDNA Reverse Transcription kit (Applied Biosystems, Foster City, CA, USA). qRT-PCR was performed in ABI Prism 7300 (Applied Biosystems) using SYBR Select Master Mix containing SYBR GreenE dye (Applied Biosystems). The relative quantity of cDNA was estimated by ΔΔCT method and data were normalized to β-actin (ACTB). Following primers were purchased from East Port (Prague, Czech Republic): ACTB_fw: 5′-CATTGCTGACAGGATGCAGAAGG-3′, ACTB_rev: 5′-TGCTGGAAGGTGGACAGTGAGG-3′; p21_fw: 5′-CAGATCCACAGCGATATCCA-3′, p21_rev: 5′-ACGGGACCGAAGAGACAAC-3′; and p16_fw: 5′-CGTGAACATGTTGTTGAGGC-3′, p16_rev: 5′-GCAGAAGAGCTGCTACGTGA-3′. Data represent values from three independent experiments performed in 3 technical replicates.

### EdU incorporation, Click-iT reaction and FACS analysis

TRAMP-C2 and TC-1 cells were driven to senescence by DTX as described above. On the fourth day of DTX treatment, cells were incubated with 10 μM 5-ethynyl-2′-deoxyuridine (EdU) for 6 h. For Click-iT reaction cells were washed by PBS, detached by trypsin, washed by PBS again and fixed by 4% formaldehyde for 15 min. The fixed cells were washed in PBS, permeabilized by 0.2% Triton X-100 for 5 min and washed by 1% BSA/PBS. Click-iT reaction was performed with Click-iT EdU Alexa Fluor 647 Kit (Invitrogen, Carlsbad, CA, USA) according to manufacturer's instructions. After that, cells were washed twice in PBS and resuspended in fresh PBS for FACS analysis. FACS analysis was performed using an LSR II flow cytometer (BD Biosciences) and data analyzed by FlowJo 7.6.5 software.

### FACS analysis of explanted tumor cells

Cell surface expression of CD45 and CD80 on the tumor cells explanted from tumor bearing mice was analyzed by flow cytometry. Cell suspensions were washed and preincubated with anti-CD16/CD32 antibody to minimize non-specific binding for 15 min at 4°C following washing step and incubation with labeled primary antibody for 30 min at 4°C. Relevant isotype controls of irrelevant specificity were used. FACS buffer (PBS, 1% FBS, 0.1% NaN_3_) was used for all washing steps and analysis. The following antibodies were used: PE anti-CD80 (16-10A1), PE anti-CD86 (GL1), BV421 anti-CD45 (30-F11) (BD Biosciences, San Jose, CA); FACS analysis was performed using an LSR II flow cytometer (BD Biosciences) and data analyzed by FlowJo 7.6.5 software.

### Immunolabeling and fluorescence microscopy

Cells were grown on glass coverslips were driven to senescence by DTX as described above. On the fourth day of DTX treatment, cells were fixed by 4% formaldehyde for 20 min and permeabilized by 0.2% Triton X-100 for 5 min. Subsequently, cells were washed with PBS, blocked in 25% FBS/PBS for 30 min and stained with primary antibodies diluted in 1% BSA/PBS for 1 h. After double-wash by PBS cells were stained with secondary antibody diluted in 1% BSA/PBS for 45 min, double-washed by PBS again and mounted in Mowiol, containing 4′,6-diamidine-2-phenylindole (DAPI; Sigma) counter-stain (1 mg/ml). Fluorescence microscopy was performed using fluorescence microscope Leica CTR6000 (Leica Microsystems) equipped with monochrome digital camera DFC350 FX and Leica LAS AF Lite software.

The antibodies used: phospho-Ser139 H2AX, rabbit, polyclonal, Cell Signaling, 1 : 200; Alexa 488 goat anti-rabbit, Invitrogen, 1 : 1000.

### Cytokine array

Cells were seeded into 75 cm^2^ flasks (1 – 3 × 10^6^ cells/flask) and after 6 h, DTX was added to a final concentration of 7.5 μM and cells were cultivated for 96 h with/without DTX (7.5 μM). Expression of selected chemokines/cytokines by tumor cells was analyzed using Mouse Cytokines & Chemokines PCR Array (PAMM-150ZG-4, QIAGEN) according to the manufacturer's instructions.

### Mice

C57BL/6 male mice, 6 - 8 weeks old, were obtained from AnLab Co., Prague, Czech Republic. Experimental protocols were approved by the Institutional Animal Care Committee of the Institute of Molecular Genetics, Prague.

### *In vivo* experiments

Mice (8 - 10 per group) were transplanted on day 0 s.c. with control TC-1 cells (3 × 10^4^), DTX-induced senescent TC-1/DTX or TRAMP-C2/DTX cells (3 × 10^5^) or control TC-1 cells (3 × 10^4^) admixed with TC-1/DTX or TRAMP-C2/DTX cells. For control, irradiated TC-1 cells (150 Gy), TRAMP-C2 cells injected in admixture with TC-1 cells were used. To characterize growing tumors, some tumors were explanted, cultured *in vitro* for 7 days and the surface expression of CD80 was analyzed by FACS. IL-12 producing TC-1/IL-12 cells were used in therapeutic experiments. Cells were administered on day 3 in the vicinity of transplanted control TC-1 cells and TC-1 cells admixture with TC-1/DTX senescent cells. Mice were observed twice a week and the size of the tumors was recorded. Two perpendicular diameters of the tumors were measured with a caliper and the tumor size was expressed as the tumor area (cm^2^).

### Data processing and statistical analyses

For evaluation of *in vitro* experiments, graph concerning qRT-PCR data was generated using Prism 5 (GraphPad Software, La Jolla, CA USA). qRT-PCR data represent mean ± S.D. *p*-values were calculated using student *t*-Test for two samples assuming unequal variances (Microsoft Excel 2010, Microsoft, Redmond, WA, USA). *p* < 0.05 was considered statistically significant.

For evaluation of *in vivo* experiments, Analysis of Variance (ANOVA) from the NCSS, Number Cruncher Statistical System (Kaysville, Utah, USA) statistical package was utilized. Standard deviations are indicated in the Figures.

## SUPPLEMENTARY MATERIALS


