# Supplementary material for: Tumor growth accelerated by chemotherapy-induced senescent cells is suppressed by treatment with IL-12 producing cellular vaccines
Source: Oncotarget. 2016 Jul 19;7(34):54952–64. doi: 10.18632/oncotarget.10712 (PMC5342393; doi:10.18632/oncotarget.10712)
Supplement: Supplementary file 1 [file oncotarget-07-54952-s001.docx]

**Supplementary Table S1: Cytokine and chemokine mRNA levels of TC-1 and TRAMP-C2 cell lines treated with DTX**

|  |  | TC-1 | TRAMP-C2 |
| --- | --- | --- | --- |
| Gene | Description | Fold change* | Fold change* |
| Csf3 | Colony stimulating factor 3 (granulocyte) | 22.94 | 5.94 |
| Tnfsf13b | Tumor necrosis factor (ligand) superfamily, member 13b | 12.21 | 7.89 |
| Csf2 | Colony stimulating factor 2 (granulocyte-macrophage) | 8.94 | 11.39 |
| Tnf | Tumor necrosis factor | 8.57 | 10.13 |
| Ccl4 | Chemokine (C-C motif) ligand 4 | 8.57 | 7.84 |
| Ccl3 | Chemokine (C-C motif) ligand 3 | 6.32 | 1.73 |
| Fasl | Fas ligand (TNF superfamily, member 6) | 6.28 | 4.66 |
| Ccl20 | Chemokine (C-C motif) ligand 20 | 5.70 | 4.96 |
| Il13 | Interleukin 13 | 5.70 | 3.63 |
| Il22 | Interleukin 22 | 5.58 | 1.73 |
| Il10 | Interleukin 10 | 5.39 | 4.92 |
| Cxcl1 | Chemokine (C-X-C motif) ligand 1 | 5.35 | 3.10 |
| Cxcl16 | Chemokine (C-X-C motif) ligand 16 | 4.76 | 3.53 |
| Hc | Hemolytic complement | 4.69 | 13.74 |
| Bmp2 | Bone morphogenetic protein 2 | 4.59 | 1.33 |
| Nodal | Nodal | 4.32 | 1.87 |
| Cxcl10 | Chemokine (C-X-C motif) ligand 10 | 4.14 | 18.00 |
| Il27 | Interleukin 27 | 3.84 | 2.30 |
| Adipoq | Adiponectin, C1Q and collagen domain containing | 3.76 | 7.84 |
| Ccl5 | Chemokine (C-C motif) ligand 5 | 3.66 | 2.97 |
| Il1a | Interleukin 1 alpha | 3.53 | 1.28 |
| Cd70 | CD70 antigen | 2.89 | 2.14 |
| Il17f | Interleukin 17F | 2.73 | 2.95 |
| Ifng | Interferon gamma | 2.68 | 1.73 |
| Lif | Leukemia inhibitory factor | 2.66 | 5.28 |
| Cxcl11 | Chemokine (C-X-C motif) ligand 11 | 2.66 | 1.73 |
| Ccl22 | Chemokine (C-C motif) ligand 22 | 2.62 | 1.73 |
| Bmp6 | Bone morphogenetic protein 6 | 2.58 | −1.27 |
| Il3 | Interleukin 3 | 2.48 | 1.73 |
| Il6 | Interleukin 6 | 2.45 | 3.10 |
| Cntf | Ciliary neurotrophic factor | 2.40 | 2.36 |
| Ccl19 | Chemokine (C-C motif) ligand 19 | 2.35 | 5.46 |
| Il1b | Interleukin 1 beta | 2.14 | 1.73 |
| Il4 | Interleukin 4 | 1.97 | −1.35 |
| Il23a | Interleukin 23, alpha subunit p19 | 1.88 | 16.00 |
| Il12a | Interleukin 12A | 1.88 | 7.41 |
| Bmp7 | Bone morphogenetic protein 7 | 1.88 | 1.73 |
| Il24 | Interleukin 24 | 1.78 | 4.89 |
| Ifna2 | Interferon alpha 2 | 1.78 | 3.05 |
| Cxcl13 | Chemokine (C-X-C motif) ligand 13 | 1.69 | 25.63 |
| Ccl2 | Chemokine (C-C motif) ligand 2 | 1.51 | 4.72 |
| Cxcl3 | Chemokine (C-X-C motif) ligand 3 | 1.32 | 5.98 |
| Il1rn | Interleukin 1 receptor antagonist | 1.29 | 4.08 |
| Il5 | Interleukin 5 | 1.23 | 5.39 |
| Il7 | Interleukin 7 | 1.14 | −1.62 |
| Tnfsf11 | Tumor necrosis factor (ligand) superfamily, member 11 | 1.13 | −1.15 |
| Ltb | Lymphotoxin B | 1.12 | 1.69 |
| Pf4 | Platelet factor 4 | 1.10 | 8.63 |
| Xcl1 | Chemokine (C motif) ligand 1 | 1.10 | 8.17 |
| Cxcl9 | Chemokine (C-X-C motif) ligand 9 | 1.10 | 7.06 |
| Ccl12 | Chemokine (C-C motif) ligand 12 | 1.10 | 6.02 |
| Ppbp | Pro-platelet basic protein | 1.10 | 3.68 |
| Osm | Oncostatin M | 1.10 | 2.99 |
| Ccl1 | Chemokine (C-C motif) ligand 1 | 1.10 | 2.58 |
| Tnfrsf11b | Tumor necrosis factor receptor superfamily, member 11b | 1.10 | 1.93 |
| Ccl24 | Chemokine (C-C motif) ligand 24 | 1.10 | 1.73 |
| Cd40lg | CD40 ligand | 1.10 | 1.73 |
| Il17a | Interleukin 17A | 1.10 | 1.73 |
| Il2 | Interleukin 2 | 1.10 | 1.73 |
| Il21 | Interleukin 21 | 1.10 | 1.73 |
| Il9 | Interleukin 9 | 1.10 | 1.73 |
| Mstn | Myostatin | 1.10 | 1.73 |
| Il12b | Interleukin 12B | 1.10 | 1.69 |
| Tgfb2 | Transforming growth factor, beta 2 | 1.10 | −1.09 |
| Mif | Macrophage migration inhibitory factor | 1.05 | 2.73 |
| Vegfa | Vascular endothelial growth factor A | −1.04 | 1.53 |
| Lta | Lymphotoxin A | −1.08 | 1.73 |
| Il11 | Interleukin 11 | −1.09 | 1.10 |
| Spp1 | Secreted phosphoprotein 1 | −1.14 | −4.00 |
| Cxcl5 | Chemokine (C-X-C motif) ligand 5 | −1.19 | 4.92 |
| Ccl11 | Chemokine (C-C motif) ligand 11 | −1.21 | 3.76 |
| Il15 | Interleukin 15 | −1.25 | 2.99 |
| Thpo | Thrombopoietin | −1.32 | 1.06 |
| Ctf1 | Cardiotrophin 1 | −1.37 | 7.62 |
| Cx3cl1 | Chemokine (C-X3-C motif) ligand 1 | −1.56 | −2.22 |
| Csf1 | Colony stimulating factor 1 (macrophage) | −1.58 | 1.39 |
| Bmp4 | Bone morphogenetic protein 4 | −1.84 | 1.32 |
| Gpi1 | Glucose phosphate isomerase 1 | −1.93 | 1.28 |
| Il18 | Interleukin 18 | −2.09 | 3.71 |
| Tnfsf10 | Tumor necrosis factor (ligand) superfamily, member 10 | −2.22 | 4.56 |
| Ccl7 | Chemokine (C-C motif) ligand 7 | −2.79 | 1.28 |
| Il16 | Interleukin 16 | −2.95 | 3.36 |
| Cxcl12 | Chemokine (C-X-C motif) ligand 12 | −2.97 | 3.94 |
| Ccl17 | Chemokine (C-C motif) ligand 17 | −4.03 | −1.45 |

*Fold change: treated cells vs. untreated cells.
